# Functional recovery with peripheral nerve block versus general anesthesia for upper limb surgery: a systematic review

**DOI:** 10.1186/s12871-023-02038-8

**Published:** 2023-03-24

**Authors:** Jennifer Héroux, Pierre-Olivier Bessette, Emilie Belley-Côté, Daphnée Lamarche, Pablo Échavé, Marie-Josée Loignon, Nicolas Patenaude, Jean-Patrice Baillargeon, Frédérick D’Aragon

**Affiliations:** 1 Department of Anesthesiology, Université de Sherbrooke, Sherbrooke, QC Canada; 2 Divisions of Cardiology and Critical Care, Department of Medicine, McMaster University, Hamilton, ON Canada; 3 Population Health Research Institute, Hamilton, ON Canada; 4 Department of Orthopedic Surgery, Université de Sherbrooke, Sherbrooke, QC Canada; 5 Division of Endocrinology, Department of Medicine, Université de Sherbrooke, Sherbrooke, QC Canada; 6 Centre de Recherche du Centre Hospitalier Universitaire de Sherbrooke, Sherbrooke, QC Canada

**Keywords:** Upper limb surgery, Peripheral anesthesia, Nerve block, Brachial plexus, Postoperative recovery, Functional recovery

## Abstract

**Background:**

Peripheral nerve block is a common anesthetic technique used during orthopedic upper limb surgery. Injection of local anesthetics around the target nerve inhibits the action of voltage-dependent sodium channels, inhibiting neurotransmission of pain impulses and providing motor immobility. Compared to general anesthesia, it could improve functional recovery by inhibiting nociceptive impulses and inflammation, thus reducing postoperative pain and immobilization and improving postoperative rehabilitation. This systematic review evaluates the impact of peripheral nerve block versus general anesthesia on postoperative functional recovery following orthopedic upper limb surgery.

**Methods:**

We searched CENTRAL, MEDLINE, CINHAL, EMBASE, and Scopus trial databases from inception until September 2021 for studies comparing peripheral nerve block to general anesthesia. We collected data on functional recovery, range of motion, patient satisfaction, quality of life, and return to work. We pooled studies using a random-effects model and summarized the quality of evidence with the GRADE approach.

**Results:**

We assessed 373 citations and 19 full-text articles for eligibility, and included six studies. Six studies reported on functional recovery, but failed to detect a significant superiority of peripheral nerve block over general anesthesia (3 RCT studies, *N* = 160; SMD -0.15; CI at 95% -0.60–0.3; I^2^ = 45%; *p* = 0.07; low quality of evidence and 3 observational studies, *N* = 377; SMD -0.35; CI at 95% -0.71–0.01; I^2^ = 64%; *p* = 0.06; very low quality of evidence).

**Conclusions:**

Current literature is limited and fails to identify the benefit of peripheral nerve block on functional recovery. More studies are needed to assess the impact on long-term recovery. Considering the potential impact on clinical practice and training, a prospective study on functional recovery is ongoing (NCT04541745).

**Trial registration:**

PROSPERO ID CRD42018116298. Registered on December 4, 2018.

**Supplementary Information:**

The online version contains supplementary material available at 10.1186/s12871-023-02038-8.

## Background

More than 22 million orthopedic surgeries are performed worldwide each year [1]. Orthopedic upper limb surgeries represent more than half of the surgeries performed yearly and are associated with severe postoperative pain requiring multimodal analgesia [1, 2]. Indeed, more than 70% of patients report moderate to high postoperative pain and tend to have a higher average opioid consumption 24 h postoperatively than in other surgeries [3–5]. Following upper limb surgery, postoperative inflammation and pain lead to a decreased range of motion in the operated joint and promote mechanical hyperalgesia, delaying recovery [3, 5–7]. Interestingly, about 15 to 20% of patients report a lower level of functionality in activities of daily living after orthopedic surgery of the upper limb, compared to the preoperative state [8–10]. Postoperative pain, by delaying the rehabilitation, might impact long-term functional recovery [11].

Recent studies have demonstrated that functional recovery is a multidisciplinary concept that relates to the limitations imposed by the body's muscles and joints, limitations in daily activities, and environmental and personal factors emphasizing the importance of including patient-reported outcomes to evaluate functional recovery [12–15]. Postoperative functional recovery of the operated limb can be assessed by the range of motion of the articulation, muscle strength, radiological signs of fracture union and with psychometric evaluation tools [12, 13].

Peripheral nerve block (PNB) is a technique that provides long-term analgesia while preventing side effects of general anesthesia (GA) [14, 16, 17]. In orthopedics, local anesthesia provides several advantages over GA, including improved postoperative pain management, reduced hospital time, lower hospital costs, and prevention of significant complications associated with GA [18]. The injection of the local anesthetic around the nerve inhibits the action of voltage-dependent sodium channels, thus inhibiting neurotransmission of pain impulses and providing motor immobility [19, 20]. Furthermore, local anesthetic agents have multiple anti-inflammatory mechanisms: blocking the activation of C fibres, reduction of cytokines production and blocking sympathetic nerve activity, which helps reduce postoperative pain and promote early mobilization of the limb [6].

For orthopedic lower limb surgery, it has been demonstrated that the use of PNB has a beneficial impact on functional recovery post-surgery. Indeed, the authors hypothesized that the functional recovery benefits of PNB are due to the reduction in postoperative pain scores and earlier mobilization [21–25]. However, the impact of postoperative pain and early mobilization on functional recovery has been relatively unexplored for upper limb surgery [6, 18, 24]. To investigate this gap in the literature, we performed a systematic review and meta-analysis of studies evaluating the effect of PNB compared with GA on functional recovery after upper limb surgery.

## Methods

This systematic review is reported based on the Preferred Reporting Items for Systematic Reviews and Meta-Analyses (PRISMA) statement [26] and is registered on PROSPERO (rCRD42018116298). Complete protocol details have been previously published [27].

### Search strategy

We performed a comprehensive search strategy of CENTRAL (The Cochrane Central Register of Controlled Trials), MEDLINE, CINHAL (Cumulative Index to Nursing and Allied Health Literature), Scopus and EMBASE from their inception to December 2018. Keyword search terms included: upper limb surgery, peripheral anesthesia, nerve block, brachial plexus, postoperative recovery, and functional recovery. Details on search strategy are available in Supplementary Tables 1, Additional File 1. We searched conference proceedings and abstracts presented between December 2016 and December 2018 from the following meetings: Canada Anesthesiologists’ Society Annual Meeting, World Congress on Regional Anesthesia and Pain Medicine, American Society of Anesthesiologists Annual Meeting and, Euroanesthesia and Société Française d’Anesthésie et de Réanimation. Trial registries (ClinicalTrials.gov and Who.int) were searched for ongoing and unpublished eligible studies. The search strategy was repeated in September 2021 to include more recent articles.

### Study selection

We included randomized controlled trials (RCTs) and observational studies assessing the use of PNB compared to GA for orthopedic upper limb surgery. Studies were included if the population of interest was adults (≥ 18 years old) undergoing orthopedic surgery of the upper limb (*e.g.,* total shoulder arthroscopy/arthroplasty, open reduction and internal fixation, tendon/muscle reimplantation, mass/tumour excision of soft tissues). We included studies comparing single-shot PNB anesthesia of the brachial plexus, defined as supraclavicular, infraclavicular, axillary and interscalene, to GA. We included studies assessing functional recovery with a validated upper limb psychometric tool and did not impose language restrictions or year of publication**.** We excluded studies if they were case reports or series, investigations using animal models, and no comparison between GA and PNB was performed. When a study included a proportion of patients with a concomitant PNB and GA, only the data on patients who did not receive concomitant GA were included.

### Data extraction

We performed data extraction independently and in duplicate using predefined data extraction forms. Extracted data included study title, first author, study design, baseline characteristics, details of the intervention (e.g., type of block, a dose of local anesthetic, surgery), relevant controls, and information on methodological quality for each study. In addition, we included the following outcomes: functional recovery after an upper limb surgery at each follow-up visit using the assessment tool reported by the study authors, patient satisfaction regarding the anesthetic technique used, range of motion and delay from surgery to the return to work. If available, we collected the following adverse events: nerve damage or other neurological injuries, vascular puncture, infection at the puncture site and chronic pain. Disagreements between reviewers were resolved through discussion or third-party adjudication.

### Quality assessment

#### Assessment of risk of bias for randomized controlled trials

The risk of bias was evaluated independently and in duplicate for each study. We resolved disagreements by consensus. For RCTs, we used the Cochrane Collaboration risk of bias tool for RCT [28]. We assessed the following elements: 1) random sequence generation, 2) allocation concealment, 3) blinding, 4) incomplete outcome data, 5) selective outcome reporting and 6) other sources of bias. According to specific criteria available in the Cochrane Collaboration risk of bias tool, biases were categorized as ‘low risk of bias, 'unclear risk of bias and ‘high risk of bias [28]. More information on the Cochrane Collaboration risk of bias tool for RCT is available in see Supplementary Table 2, Additional File 1. We considered studies to be ‘low risk’ if all aspects were considered to have ‘low’ risk of bias. Studies with at least one aspect considered to have ‘high’ risk of bias were considered overall to be ‘high’’ risk. We resolved disagreements by discussion or with the help of a third reviewer. Because of the limited number of studies, we did not address publication bias.

#### Assessment of risk of bias in observational studies

For observational studies, we used the Clinical Advances through Research and Information Translation (CLARITY) tools [29]. These tools assesses eight domains: 1)exposed and non-exposed cohorts were drawn from the same population; 2)confidence in the assessment of the exposure; 3) absence of an outcome of interest at the start of the study; 4) exposed and non-exposed cohorts matched for all variables or statistical adjustment; 5) confidence in the assessment of the presence or absence of prognostic factors; 6) confidence in the assessment of the outcome; 7) quality of follow-up; 8) similarity in co-interventions between the groups. For each criterion, we evaluated the risk of bias as 'definitely yes,' 'probably yes,' 'probably no,' and 'definitely no [29]. For more detail, see Supplementary Table 3, Additional File 1.

#### Assessment of confidence in pooled effect estimates

We assessed the quality of evidence of each outcome using the Grading of Recommendations, Assessment, Development and Evaluation (GRADE) framework [30]. GRADE approach evaluated five potential factors affecting the quality of evidence: 1) limitations in the design and implementation of available studies (individual study risk bias); 2) indirectness (the extent to which study population, intervention, and outcome deviate from those of interest for this systematic review); 3) inconsistency of results between studies; 4) imprecision of treatment effect, and 5) publication bias [28, 30]. Disagreements for GRADE assessments was resolved by discussion and consensus.

### Data and statistical analyses

We calculated chance-corrected agreement for relevance and eligibility decision using the kappa statistic [31]. We conducted the statistical analysis with R (R Core Team, v.4.0.2, 2021) using the ‘metafor’ package [32]. Continuous outcomes were evaluated with a standardized mean difference (SMD) and 95% confidence interval (Cl). We used SMD because functional recovery is measured in a variety of ways [33]. We used a random-effects model of DerSimonian for pooled analysis. Heterogeneity was assessed with a chi-squared test and the I^2^ statistic. In the use of multiple evaluations of functional recovery, we used the data available at the final follow-up visit. With the aim of limiting the impact of the heterogeneity and variability of the estimates on the results we also performed subgroup analysis based on the design subtype. We did not perform subgroup or sensitivity analysis.

## Results

### Study selection

**Fig. 1:**
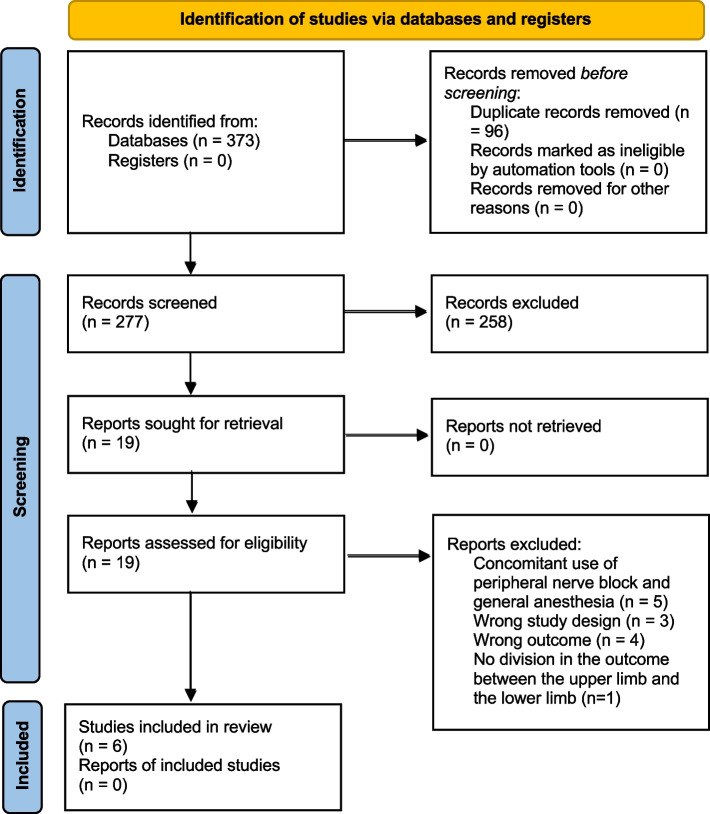
PRISMA flow chart of the screening and selected studies

We identified 373 potentially relevant citations (Fig. 1). Following the study selection, six studies were included in the systematic review [34–39]. Agreement between reviewers for relevance (k = 0.85) and eligibility (k = 1) was considered excellent. Ultimately we included three RCTs [36, 39, 40], two retrospective cohort studies [34, 35] and one prospective cohort study [37]. All studies were published in English between 2012 and 2020. There was no ongoing study comparing regional to PNB to GA on clinicaltrial.org.

### Study characteristics

Study characteristics are summarized in Table 1 and for more details see Supplementary Table 4, Additional File 1. Five studies assessed clinical outcomes of the wrist using infraclavicular block [34, 36, 37, 39, 40], and one evaluated the shoulder joint using an interscalene block approach [35]. The surgical indication was distal radial fracture fixation [34, 36, 39, 40], proximal humerus fracture fixation [35] and multiples wrist surgery (e.g. carpal tunnel release, wrist mass excision, ganglion cyst excision, metacarpal fracture Open Reduction Internal Fixation (ORIF), fracture correction, hardware removal, arthroscopic surgery and tendon/ligament repair) [37]. The lengths of follow-up were, respectively, seven days [37], three months [36], six months [39, 40] and twelve months [34, 35].

**Table 1:** Description of studies included in the systematic review

1^st^ author, Year	Study Design	Population	Surgery	Anesthetic procedure	Follow-up duration	Clinical Findings
		**N**				
**RCT**						
Galos, 2016 [36]	Monocenter	36	Distal radius fracture fixation	**Group PNB** Type: Infraclavicular nerve block Agents: Lidocaine 2% with 1:200,000 epinephrine (20 mL) and bupivacaine 0.25% (15 mL) If failed: N/A	3 months	Functional recovery: DASH, SMFA No difference between PNB and GA group ROM: N/A Time frame from surgery to return to work: N/A Satisfaction regarding the anesthesia technique used: N/A Complications: N/A
				**Group GA** Regimen at the discretion of the anesthesiologist		
Rundgren, 2019 [39]	Monocenter	90	Distal radius fracture fixation	**Group PNB** Type: Supraclavicular nerve block Agents: Mepicavaine 1% (2/3 solution) and levobupivacaine 0.25% (1/3 solution) If failed: No intention to treat analysis was done	6 months	Functional recovery: EQ/ED/3L, PWRE No difference between PNB and GA group (EQ-ED-3L *p* = 0.7; PRWE *p* = 0.7) ROM: NS difference (*p* = 0.7) Time frame from surgery to return to work: N/A Satisfaction regarding the anesthesia technique used: N/A Complications: N/A
				**Group GA** Induction: Propofol* Fentanyl* Maintenance: Sevoflurane* Fentanyl* End of surgery: Infiltration 10 mL levobupicavaine 0.5% *Dose at the discretion of the anesthesiologist		
Wong, 2020 [38]	Monocenter	52	Distal radius fracture fixation	**Group PNB** Type: Infraclavicular nerve block Agents: Lidocaine 2% 10 mL with 1:200,000 epinephrine and 10 mL of ropivacaine 0.75% If failed: No intention to treat analysis was done	6 months	Functional recovery: QuickDASH, PRWE No difference between PNB and GA group ROM: N/A Time frame from surgery to return to work: N/A Satisfaction regarding the anesthesia technique used: N/A Complications: N/A
				**Group GA** Induction: Fentanyl (0.25-2mcg/kg) Propofol (1.5-3 mg/kg) Atracurium (0.5 mg/kg) Maintenance: Sevoflurane (MAC 0.7–1.5) Morphine (0.025–0.05 mg/kg) End of surgery: Infiltration levobupicavaine 0.5% 2 mg/kg		
**Observational studies**						
Egol, 2012 [34]	Retrospective	187	Distal radius fracture fixation	**Group PNB** Type: Infraclavicular nerve block Agents: N/A If failed: N/A	12 months	Functional recovery: DASH No difference between PNB and GA group (*p* = 0.72) ROM: PNB is superior to GA group for: -wrist extension -wrist flexion -index finger total active movement -ring finger distal palmar crease Time frame from surgery to return to work: N/A Satisfaction regarding the anesthesia technique used: N/A Complications: N/A
				**Group GA** Regimen at the discretion of the anesthesiologist		
Egol 2014 [35]	Retrospective	122	Proximal humerus fracture repair	**Group PNB** Type: Interscalene Brachial plexus block Agents: N/A If failed: N/A	12 months	Functional recovery: DASH PNB is superior to GA group (*p* = 0.003) ROM: PNB is superior to GA group for: -active forward elevation (*p* = 0.002) -passive forward elevation (*p* = 0.005) -external rotation (*p* = 0.002) Time frame from surgery to return to work: N/A Satisfaction regarding the anesthesia technique used: N/A Complications: N/A
				**Group GA** Regimen at the discretion of the anesthesiologist		
Doo, 2020 [37]	Prospective	119	Fracture correction Hand ware removal Arthroscopic surgery Tendon/ligament repair Carpal tunnel release Mass excision Other	**Group PNB** Type: Supraclavicular Agents: Lidocaine 1.5% with 1:200,000 epinephrine If failed: N/A	7 days	Functional recovery: Global QoR-40 K No difference between PNB and GA group (*p* = 0.21) ROM: N/A Time frame from surgery to return to work: N/A Satisfaction regarding the anesthesia technique used: N/A Complications: N/A
				**Group GA** Induction: Propofol (1.5–2.5 mg/kg) Rocuronium (0.3–0.8 mg/kg) Maintenance: Sevoflurane (1–4%) Perfusion remifentanil (1.3 mcg/kg) End of surgery: N/A		

*DASH* Disabilities of the Arm, Shoulder and Hand, * EQ-ED-3L* EuroQol-5 Dimensions-3, *GA* General Anesthesia, *N/A* Not Available, *NS* No statistical, *ORIF* Open Reduction Internal Fixation, *PNB* Peripheral Nerve Block, *PO* Postoperative, *PWRE* Patient Rated Wrist Evaluation, *QoR-40 K* Quality of Recovery – 40 Korean, *QuickDASH* Quick Disabilities of the Arm, Shoulder and Hand, *RCT* Randomized Control Trial, *ROM* Range of Motion, *SMFA* Short Musculoskeletal Function Assessment

### Risk of bias assessment

All RCTs were categorized at high-risk-of-bias for the absence of blinding of the patient and operating staff on the anesthesia technique, PNB vs GA, and absence of blinding of the research team responsible for collection of postoperative and specific outcome data (Table 2) [37, 39, 40]. All observational studies were judged to be at high-risk-of-bias for the absence of matching of exposed and unexposed participants for variables associate with an impact on the outcome of interest, for example the type of surgery (Table 2) [34, 35, 37]. In two studies, there was an unclear risk of bias for assessment on prognostic data since the authors failed to provide information on the staff collecting the data for each outcome and for the follow-up quality since there were a lot of losses to follow-up with plausible impact on the outcome of interest [34, 35].

**Table 2:** Risk of Bias

RCT	Selection		Performance	Detection	Attrition	Reporting	Others	
	**Randomisation**	**Allocation**						
Galos, 2016 [36]								
Rundgren, 2019 [39]								
Wong, 2020 [40]								
**Observational studies**	**Exposed and non-exposed cohort population**	**Assessment exposure**	**Absence outcome before study**	**Exposed and non-exposed matched**	**Assessment outcome**	**Assessment prognostic factor**	**Follow-up quality**	**Similiarity co-intervention**
Egol, 2012 [34]								
Egol, 2014 [35]								
Doo, 2020 [20]								

Risk of Bias Graph. For RCT: Cochrane Collaboration risk of bias tool. For observational studies: CLARITY (Clinical Advances through Research and Information Translation) tool

*RCT* Randomized Control Trial

Explanations

Low risk of bias (RCT)/definitively yes (observational studies)

Unclear (RCT)/probably yes (observational studies)

High (RCT)/ definitively non (observational studies)

### Outcomes

Six studies (*N* = 563) assessed the functional recovery post upper limb surgery performed under PNB versus GA.

#### Assessment of functional recovery

**Fig. 2:**
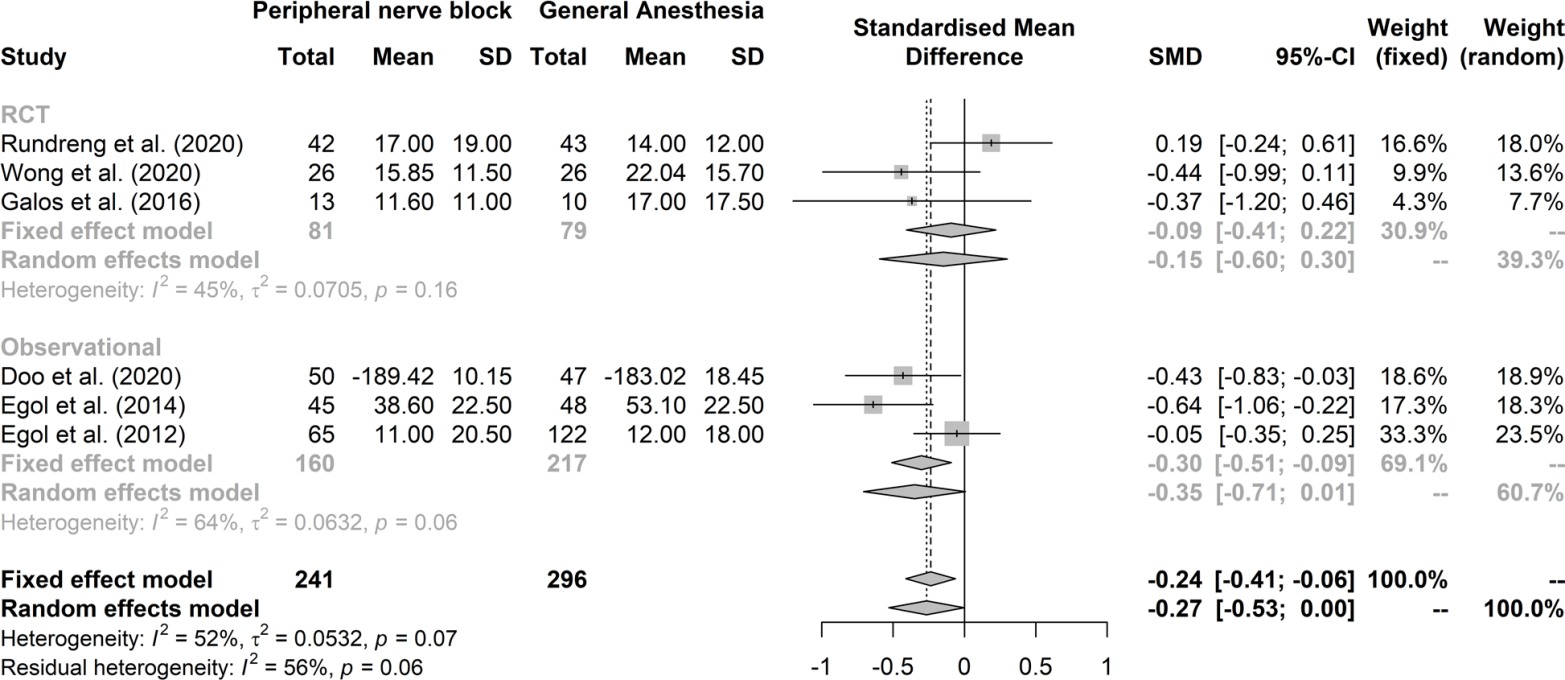
Functional recovery at the latest follow-up and maximal recovery follow-up with superiority of PNB over GA

The data for assessing functional recovery at the final follow-up were pooled (Fig. 2). For observational retrospective studies, functional recovery following upper limb surgery at the final follow-up suggests a superiority of PNB over GA with a small size effect (3 studies, *N* = 377; SMD -0.35; 95% CI -0.71–0.01; I^2^ = 64%; very low confidence). We rated the overall quality of evidence very low because of the high-risk bias aforementioned and for inconsistency and imprecision in the studies included. For RCTs, no significant difference was detected between PNB and GA for the functional recovery (3 studies, *N* = 160; SMD -0.15; CI at 95% -0.60–0.3; I^2^ = 45%; low confidence). The quality of evidence was deemed very low because of imprecision and inconsistency and the presence of multiples bias in the studies. Details on the assessment of the quality of evidence is available in Supplementary Table 4, Additional File 1.

The timeframe for assessing functional recovery at final follow-up was variable among the studies included in the review ranging from seven days to twelve months [34–37, 39, 40]. No differences between anesthetic technique on functional recovery were detected at seven days and three and six months follow-ups after wrist surgery in six studies [34, 36, 37, 39, 40]. However, Egol et al. showed that for proximal humerus fracture fixation, PNB was superior to GA for functional recovery at twelve months post-surgery (DASH questionnaire (mean)- PNB: 38.6; GA:53.1; *p* = 0.003) [35]. Furthermore, in one study, the authors demonstrated that at the follow-up visits preceding the final follow-up, functional recovery following radial distal fracture fixation with PNB was superior to GA (3 months follow-up: DASH questionnaire (mean; Standard Deviation (SD)) – PNB: 18.4 (19.6) vs GA: 26.3 (27.6); *p* = 0.04) (6 months follow-up: DASH questionnaire (mean; SD) – PNB: 10.2 (18.2) vs GA: 17.8 (20.7); *p* = 0.02) [34] Information on the assessment of functional recovery at each time frame for individual studies is available in Supplementary Table 5, Additional File 1.

#### Psychometric questionnaires

Six different psychometric questionnaires were used to assess functional recuperation post-surgery [41–46]. In one study, the QoR-40 K questionnaire was used for the postoperative period and found no difference between the anesthesia technique and the recovery at seven days [37]. At twelve weeks, one study used both the DASH and SFMA questionnaire and found no difference between PNB and GA with both scores [36]. Six months post-surgery, two studies used, respectively, the PRWE and DASH questionnaires the PRWE and EQ-ED-3L questionnaires and failed to identify a difference between the type of anesthesia and the results of the psychometric questionnaires [39, 40]. At twelve months, two studies assessed functional recovery with the DASH questionnaire and found no difference between PNB and GA groups [34, 35]. All questionnaires aforementioned have been previously validated [43–48].For more information psychometrics properties of each questionnaire, see Supplementary Table 6, Additional File 1.

#### Range of motion

Three RCTs assessed the patient’s range of motion ROM [34, 35, 39]. The timeframe for evaluating ROM at final follow-up was between six months and twelve months [34, 35, 39]. Two studies evaluated the wrist and hand ROM [34, 39], and one evaluated the shoulder ROM [35]. Due to the difference in the articulation studied (shoulder versus wrist) and the different range of motion measured in each study, data could not be pooled. ROM results are available in Supplementary Table 7, Additional File 1.

#### Patient satisfaction

No study assessed satisfaction concerning the anesthesia technique or the time frame between surgery and the return to work.

#### Adverse events

Three studies evaluated peripheral nerve block related adverse events. There was no difference of neurological complications associated with the type anesthesia [35, 49, 50].

## Discussion

We systematically reviewed six studies comparing the impact of the type of anesthesia (PNB vs GA) on functional recovery after upper limb surgery. Individual studies used different types of PNB and assess various study outcomes. Our initial hypothesis was that PNB would improve postoperative functional recovery in comparison to GA since the reduction in postoperative pain and early mobilization had an impact on functional recovery for the lower limb [21–25]. Very few studies suggested any difference between PNB and GA for functional recovery [34, 35] and the pooled results did not support a treatment effect. Several factors could explain the lack of association between the type of anesthesia for upper limb orthopedic surgery and functional recovery.

The first potential explanation is the great heterogeneity observed within the studies included in this systematic review. The most striking elements explaining this clinical heterogeneity are the variability within the methodology for the assessment of clinical outcomes, the surgical indication, the joint involved and the timing of the follow-up of the participants between the studies included in this review. We had four self-reported clinical questionnaires for six studies assessing different aspects of the postoperative phase [34–37, 39, 40]. Certain questionnaires focused on carrying out daily activities and pain, such as the DASH, the QuickDASH and the PWRE questionnaire [41, 42, 44], while others included emotional aspects related to postoperative recoveries, such as SFMA, the Global QoR-40 K and the EQ-ED-3L [43, 46, 51]. Consequently, it is challenging to compare the score obtained from various questionnaires and the functional recovery may not be generalizable between studies due to the lack of standardization in the field [52]. Furthermore, the surgical indication between studies were variables including acute condition (*e.g.* fracture), minor condition (*e.g.* cyst excision) or chronic indication (*e.g.* carpal tunnel release, material removal, tendon/ligament repair) [34–37, 39, 40]. The impact of the surgical indication is essential to consider because the pain level, duration, and recovery time are not equivalent to a carpal tunnel release versus open reduction internal fixation (ORIF) [11, 53]. Moreover, the level of preoperative functionality is not the same for an acute condition versus a chronic condition [47, 54, 55]. Indeed, in terms of preoperative scores, a patient with a chronic condition will have higher disability scores [47, 54, 55]. This difference may result in the persistence of a higher postoperative disability score and would result in a decrease in the strength of association between the type of anesthesia and functional recovery. However, no corrections were made in this study to assess this limitation since, in one study, acute and chronic conditions were jointly assessed [37]. Finally, there was a difference between studies in the joint involved and the duration of the follow-up period. Five studies [34, 36, 37, 39, 40] assessed the wrist, while one study assessed the shoulder [35], and the duration of patient follow-up lasted from seven days to twelve months [34–37, 39, 40]. An essential element to consider is the expected duration of the postoperative recovery phase depending on the type of surgery, which is three months for the wrist and six to twelve months for the shoulder [56]. Thus, considering the joint involved, the time frame between the surgery and the evaluation of functional recovery could impact the results and be a confounding factor. In the statistical analysis, this would decrease the strength of the association between the PNB and functional recovery, contributing to the non-statistical difference. Considering that we hypothesized that the use of locoregional anesthesia decreases postoperative pain and inflammation, comparing different surgery types could have impacted the results obtained with the psychometric questionnaires and consequently limit the possible comparison.

The studies were included in the review and had great variability in the study design and the presence of multiples bias. To limit the impact of this factor, we analyzed by study design subtype since three studies were RCTs [37, 39, 40], and three were observational studies [34–36]. Despite this, the heterogeneity coefficients remained high, indicating that the sources of heterogeneity were multiple. Indeed, the lack of blinding of the participants and staff collecting the data, the important loss of participants in one study [36], the variability in the time frame follow-up of the participants and the low number of total events contributed to the low and the very low quality of evidence of the results in this review.

The second potential explanation for the absence of difference between PNB and GA on functional recovery is the low number of studies included in this review. We excluded surgery performed under a combined anesthesia technique (PNB and GA). The reason behind this methodological choice was to determine the effectiveness of the PNB for the perioperative period and thus ensure the validity of the data taken postoperatively. We also excluded the technique with the catheter placement versus a single-shot technique. Moreover, due to the novelty of using functional recovery for postoperative assessment of orthopedic surgery, a limited number of studies were identified. Interestingly, only six studies were published on this topic since the last narrative review, which was ten years ago [21].

The final potential explanation for the lack of association is the possibility that there is no clinical association between PNB and postoperative functional recovery. However, we respectfully believe that there are several factors going against this hypothesis. First, *Egol *et al*.*, in two independent studies, demonstrate an association between PNB and functional recovery using the DASH questionnaire and the ROM [34, 35]. Indeed, for functional recovery post distal radius fracture fixation, there was an association between PNB and functional recovery at the three-month and six-month follow-ups [34]. However, there was no difference at twelve months post-surgery, which was the last follow-up and the data used in the statistical analysis [34] The implication is that PNB might favour functional recovery in the initial recovery period, but has no impact on long-term. Second, as previously mentioned, the association between PNB and postoperative functional recovery is more established for the lower limb [21–25]. Indeed, there is a higher number of studies investigating lower limb surgeries resulting in a greater number of participants, and studies are using standardized functional recovery assessment tools between the studies [21–25].

This systematic review highlights three challenges of research in the field of PNB and functional recovery following upper limb orthopedic surgery: duration of recovery according to the joint involved (i.e., wrist, elbow, shoulder) involved, type of surgery performed, as well as the variability and the complexity of assessing functional recovery. Thus, we suspect/hypothesize that by normalizing questionnaires and surgery indications we could reduce the heterogeneity and optimize the assessment of functional recovery in this population. Indeed, in a prospective study, we are currently investigating the functional recovery of upper limb surgery with multiple psychometrics tools (NCT04541745).

## Conclusions

Current studies do not support the benefits of PNB for patients undergoing upper limb surgery. However, the low quality of evidence and high heterogenicity in the studies make it insufficient to rule out the possibility of benefits concerning functional recovery. Consequently, future studies evaluating functional recovery following upper limb surgery under PNB using appropriate psychometric evaluation and physical examination are needed.

## Supplementary Information

**Additional file 1: Table S1.** Research Strategy. **Table S2.** Cochrane Collaboration risk of bias tool for RCT. **Table S3.** CLARITY for Cohort studies. **Table S4.** Quality of Evidence. **Table S5.** Functional recovery at individual timeframe. **Table S6.** Psychometric questionnaires. **Table S7.** ROM at individual timeframe.

## Data Availability

The datasets analysed during the current study available from the corresponding author on reasonable request.

## Abbreviations

CENTRAL: The Cochrane Central Register of Controlled Trials
CINHAL: Cumulative Index to Nursing and Allied Health Literature
DASH: Disabilities of the Arm, Shoulder and Hand
EQ-ED-3L: EuroQol-5 Dimensions-3
GA: General Anesthesia
N/A: Not Available
NS: No statistical
ORIF: Open Reduction Internal Fixation
PNB: Peripheral Nerve Block
PO: Postoperative
PWRE: Patient Rated Wrist Evaluation
QoR-40 K: Quality of Recovery – 40 Korean
QuickDASH: Quick Disabilities of the Arm, Shoulder and Hand
RCT: Randomized Control Trial
ROM: Range of Motion
SG: Standard Deviation
SMFA: Short Musculoskeletal Function Assessment
