# Supplementary material for: Functional recovery with peripheral nerve block versus general anesthesia for upper limb surgery: a systematic review
Source: BMC Anesthesiol. 2023 Mar 24;23:91. doi: 10.1186/s12871-023-02038-8 (PMC10037794; doi:10.1186/s12871-023-02038-8)
Supplement: Supplementary file 1 — Additional file 1: Table S1. Research Strategy. Table S2. Cochrane Collaboration risk of bias tool for RCT. Table S3. CLARITY for Cohort studies. Table S4. Quality of Evidence. Table S5. Functional recovery at individual timeframe. Table S6. Psychometric questionnaires. Table S7. ROM at individual timeframe. [file 12871_2023_2038_MOESM1_ESM.docx]

**Functional Recovery with Peripheral Nerve Block Versus General Anesthesia for Upper Limb Surgery: A Systematic Review**

Jennifer Héroux, MD^1^, Pierre-Olivier Bessette, MD^1^, Emilie Belley-Côté, MD^2,3^, Daphnée Lamarche^1^, Pablo Échavé, MD^1^, Marie-Josée Loignon, MD^1^, Nicolas Patenaude, MD^4^, Jean-Patrice Baillargeon, MD^5,6^ and Frédérick D’Aragon, MD^1,6^

**ADDITIONAL FILE 1**

**SUPPLEMENTARY TABLES**

**Table S1:** **Research Strategy**

| P | "Upper limb surger*" OR  "Shoulder surger*" OR  "Elbow surger*" OR  "Arm surger*" OR  "Wrist surger*" OR  "Hand surger*" OR  "Finger surger*" OR  "Radius surger*" OR  "Humerus surger*" OR  "Acromion surger*" OR  "Upper limb procedure*" OR  "Shoulder procedure*" OR  "Elbow procedure*" OR  "Arm procedure*" OR  "Hand procedure*" OR  "Wrist procedure*" OR  "Finger procedure*" OR  "Humerus procedure*" OR  "Radius procedure*" OR  "Acromion procedure*" OR  "Upper Extremity Surger*" OR  "Orthopedic Procedure*" OR  Arthroscop* OR  "Upper limb fractur*" OR  "Shoulder fractur *" OR  "Elbow fractur *" OR  "Arm fractur *" OR  "Wrist fractur*" OR  "Hand fractur*" OR  "Finger fractur*" OR  "Radius fractur*" OR  "Humerus fractur*" OR  "Acromion fractur*" OR  "Upper limb fixatio*" OR  "Shoulder fixatio*" OR  "Elbow fixatio *" OR  "Arm fixatio *" OR  "Wrist fixatio*" OR  "Hand fixatio *" OR  "Finger fixatio *" OR  "Radius fixatio *" OR  "Humerus fixatio*" OR  "Acromion fixatio*" OR |
| --- | --- |
|  | MeSH: exp Upper Extremity/su [Surgery] OR Orthopedic Procedures OR Arthroscopy |
| I | "Local anaesthesi*" OR  "Local anesthesi*" OR  "Regional anesthesia" OR  "Regional anaesthesia" OR  "Peripheral anesthesia" OR  "Peripheral anaesthesia" OR  "Nerve block*" OR  "brachial plexus" OR  "Supraclavicular block*" OR  "Axillary block*" OR  "Infraclavicular block*" OR  "interscalene block*" OR |
|  | MeSH: anesthesia, local OR nerve block OR brachial plexus block |
| C | "General anesthesia" OR  "General anaesthesia" OR |
|  | MeSH: Anesthesia, General |
| 0 | "Postoperative recovery" OR  "Postopertative motion" OR  "Postoperative functional" OR  "Functional recovery" OR  "Quality of Life" OR  "treatment outcome*" OR |
|  | MeSH: "Quality of Life" OR treatment outcome |

**Table S2: Cochrane Collaboration risk of bias tool for RCT**

| REF ID: | | | | | | | | | | |
| --- | --- | --- | --- | --- | --- | --- | --- | --- | --- | --- |
| Domain | Description | | | High risk of bias | | Low risk of bias | | Unclear risk of bias | Reviewer Assessment | |
| Selection bias  Random sequence generation | Described the method used to generate the allocation sequence in sufficient detail to allow an assessment of whether it should produce comparable groups.  Reviewer Comments: | | | Selection bias (biased allocation to interventions) due to inadequate generation of a randomized sequence. | | Random sequence generation method should produce comparable groups | | Not described in sufficient detail | Judgement  Random sequence generation   - High - Low - Unclear | |
| Selection bias  Allocation concealment | Described the method used to conceal the allocation sequence in sufficient detail to determine whether intervention allocations could have been foreseen in advance of, or during, enrollment.  Reviewer Comments: | | | Selection bias (biased allocation to interventions) due to inadequate concealment of allocations prior to assignment. | | Intervention allocations likely could not have been foreseen in advance of, or during, enrollment | | Not described in sufficient detail | Judgement  Allocation concealment   - High - Low - Unclear | |
| Reporting bias  Selective reporting | Stated how the possibility of selective outcome reporting was examined by the authors and what was found.  Reviewer Comments: | | | Reporting bias due to selective outcome reporting. | | Selective outcome reporting bias not detected | | Insufficient information to permit judgement (It is likely that the majority of studies will fall into this category.) | Judgement  Selective reporting   - High - Low - Unclear | |
| Other bias  Other sources of bias | Any important concerns about bias not addressed above. If particular questions/entries were pre-specified in the study’s protocol, responses should be provided for each question/entry.  Reviewer Comments: | | | Bias due to problems not covered elsewhere in the table. | | No other bias detected | | There may be a risk of bias, but there is either insufficient information to assess whether an important risk of bias exists; or insufficient rationale or evidence that an identified problem will introduce bias. | Judgement  Other sources of bias   - High - Low - Unclear | |
| Performance bias  Blinding (participants and personnel) | | Described all measures used, if any, to blind study participants and personnel from knowledge of which intervention a participant received. Provided any information relating to whether the intended blinding was effective. | Performance bias due to knowledge of the allocated interventions by participants and personnel during the study. | | Blinding was likely effective. | | Not described in sufficient detail | | | Judgement  Blinding (participants and personnel)   - High - Low - Unclear |
|  | | Reviewer Comments: |  | |  | |  | | |  |
| Detection bias  Blinding (outcome assessment) | | Described all measures used, if any, to blind outcome assessors from knowledge of which intervention a participant received. Provided any information relating to whether the intended blinding was effective. | Detection bias due to knowledge of the allocated interventions by outcome assessors. | | Blinding was likely effective. | | Not described in sufficient detail | | | Judgement  Blinding (outcome assessment)   - High - Low - Unclear |
|  | | Reviewer Comments: |  | |  | |  | | |  |
| Attrition bias  Incomplete outcome data | | Described the completeness of outcome data for each main outcome, including attrition and exclusions from the analysis. Stated whether attrition and exclusions were reported, the numbers in each intervention group (compared with total randomized participants), reasons for attrition/exclusions where reported. | Attrition bias due to amount, nature or handling of incomplete outcome data. | | Handling of incomplete outcome data was complete and unlikely to have produced bias | | Insufficient reporting of attrition/exclusions to permit judgment of ‘Low risk’ or ‘High risk’ (e.g. number randomized not stated, no reasons for missing data provided) | | | Judgement  Incomplete outcome data   - High - Low - Unclear |
|  | | Reviewer Comments: |  | |  | |  | | |  |

**Table S3: CLARITY for Cohort studies**

| Description | High risk of bias | Higher risk of bias | Low risk of bias | Reviewer Assessment |
| --- | --- | --- | --- | --- |
| Was selection of exposed and non-exposed cohorts drawn from the same population?  Reviewer Comments: | Example:  Exposed and unexposed presenting to different points of care over a different time frame |  | Example:  Exposed and unexposed drawn for same administrative data base of patients presenting at same points of care over the same time frame | Judgement   - Definitely yes   (low risk of bias)   - Propably yes - Propably no - Definitely no   (high risk of bias) |
| Can we be confident in the assessment of the exposure?  Reviewer Comments: | Example:  Uncertain how exposure information obtained | Example:  Structured interview at a single point in time  Written self report  Individuals who are asked retrospectively confirm their exposure status mauy be subject to recall bias | Example:  Secure record (ex: surgical records)  Repeated interview or other ascertainment asking about current use/exposure | Judgement   - Definitely yes   (low risk of bias)   - Propably yes - Propably no - Definitely no   (high risk of bias) |
| Can we be confident that the outcome of interest was not present at start of study?  Reviewer Comments: |  |  |  | Judgement   - Definitely yes   (low risk of bias)   - Propably yes - Propably no - Definitely no   (high risk of bias) |
| Did the study match exposed and unexposed for all variables that are associated with the outcome of interest or did the statistical analysis adjust for these prognostic variables?  Reviewer Comments: | Example:  Matching or adjustment for a minority of plausible prognostic variables  No matching or adjustment of plausible prognostic variables  Statements of no difference between groups  Statements that differences were not statistically significant are not sufficient for establishing comparability | Example:  Matching or adjustment for most plausible prognostic variables | Example:  Comprehensive matching or adjustment for all plausible prognostic variables | Judgement   - Definitely yes   (low risk of bias)   - Propably yes - Propably no - Definitely no   (high risk of bias) |
| Can we be confident in the assessment of the presence or absence of prognostic factors?  Reviewer Comments: | Example:  Prognostic information from data base with no available documentation of quality of abstraction of prognostic variables | Example:  Chart review without demonstration of reproducibility  Data base with uncertain quality of abstraction of prognostic information | Example:  Interview of all participants  Self-completed survey from all participants  Review of charts with reproducibility demonstrated  Form data base with documentation of accuracy of abstraction of prognostic data | Judgement   - Definitely yes   (low risk of bias)   - Propably yes - Propably no - Definitely no   (high risk of bias) |
| Can we be confident in the assessment of the outcome?  Reviewer Comments: | Example:  Uncertain (no description) | Example:  Independent assessment unblinded  Self-report  For some outcomes reference to the medical record would not be adequate outcomes | Example:  Independent blind assessment  Record linkage  For some outcomes,(ex: fracture) reference to the medical record I sufficient to satisfy the requirement for confirmation of the fracture | Judgement   - Definitely yes   (low risk of bias)   - Propably yes - Propably no - Definitely no   (high risk of bias) |
| Was the follow up of cohort adequate?  Reviewer Comments: | Example:  Reason for missing outcome data likely to be related to true outcome, with either imbalance in numbers or reasons for missing data across intervention groups  For dichotomous outcome data, the proportion of missing outcomes compared with observed event risk is enough to induce important bias in intervention effect estimate  For continuous outcome data, plausible effect size (difference in means or standardized difference in means) among missing outcomes is large enough to induce clinically relevant bias in the observed effect size |  | Example:  No missing outcome data  Reasons for missing outcome data unlikely to be related to true outcome (for survival data, censoring is unlikely to introduce bias)  Missing outcome data balanced in numbers across intervention groups, with similar reasons for missing data across groups  For dichotomous outcome data, the proportion of missing outcomes compared with observed event risk is not enough to have an important impact on the intervention effect estimate  For continuous outcome data, plausible effect size (difference in means or standardized difference in means) among missing outcomes is not large enough to have an important impact on the observed effect size  Missing data have been imputed using appropriated methods | Judgement   - Definitely yes   (low risk of bias)   - Propably yes - Propably no - Definitely no   (high risk of bias) |
| Were co-interventions similar between groups?  Reviewer Comments: | Example:  Few or no relevant co-interventions that might influence the outcome of interest are documented to be similar in the exposed and unexposed |  | Example:  Most or all relevant co-interventions that might influence the outcome of interest are documented to be similar in the exposed and unexposed | Judgement   - Definitely yes   (low risk of bias)   - Propably yes - Propably no - Definitely no   (high risk of bias) |

**Table S4: Quality of Evidence**

| **Certainty assessment** | | | | | | | **Number of patients** | | **Effect** | **Certainty** | **Importance** |
| --- | --- | --- | --- | --- | --- | --- | --- | --- | --- | --- | --- |
| Number of studies | Study design | Risk of bias | Inconsistency | Indirectness | Imprecision | Other considerations | PNB | GA | Absolute  (95% CI) |  |  |
| **Functional recovery – RCT (follow-up: range 3 months to 6 months; assessed with: multiples psychometrics tools)** | | | | | | | | | | | |
| 3 | RCT | Very serious^a,b,c^ | Serious^d^ | Not serious | Serious^e,f^ | strong association; all plausible residual confounding would reduce the demonstrated effect | 81 | 79 | SMD **0.15 SD lower**  (0.6 lower to 0.3 higher) | ⨁⨁◯◯  Low | CRITICAL |
| **Functional recovery – observational studies (follow-up: range 7 days to 1 year; assessed with: multiples psychometric tools)** | | | | | | | | | | | |
| 3 | Observational | Very serious^g,h,i^ | Very serious^d,j^ | Not serious | Serious^e,f^ | strong association; all plausible residual confounding would reduce the demonstrated effect | 160 | 217 | SMD **0.35 SD lower**  (0.71 lower to 0.01 higher) | ⨁◯◯◯  Very low | CRITICAL |

Legend:

CI: Confidence Interval

GA: General Anesthesia

PNB: Peripheral Nerve Block

RCT: Randomized Control Trial

RD: Risk Difference

SMD: Standardised Mean Difference

Explanations

a. Performance bias: patient, operating room and recovery room staff and anesthesiologist were not blinded

b. Detection bias: staff collecting data were not blinded

c. Attrition bias: loss of participants important in one of the studies

d. Differences in the time frame follow-up of the participants

e. A low number of total events

f. large confidence interval

g. Exposed and non-exposed were not matched

h. Lack of information on the assessment of prognostic details

i. Lack of information on follow-up quality

j. The difference in the articulation evaluated: shoulder, wrist or forearm

k. The study did not assess the primary outcome: functional recovery

**Table S5: Functional recovery at individual timeframe**

| **1^st^ Author, Year** | **Functional Recovery** | |
| --- | --- | --- |
| **RCT** | | |
| Galos (1),  2016 | 6 weeks: DASH score (mean (SD))  PNB: 26.7 (25.6)  GA: 33.7 (19.4)  No difference between PNB and GA group (p=0.408)  12 weeks: DASH score (SD), mean  PNB: 11.6 (11)  GA: 17 (17.5)  No difference between PNB and GA group (p=0.377) | 6 weeks: SMFA score (SD), mean  Function:  PNB 29.3 (29.1)  GA 29.7 (20.7)  No difference between PNB and GA group (p=0.956)  Bothersome:  PNB: 19.1 (23.2)  GA: 18.3 (17.0)  No difference between PNB and GA group (p=0.915)  Activity:  PNB: 25.1 (25.9)  GA: 25.3 (18.6)  No difference between PNB and GA group (p=0.982)  Emotion:  PNB: 20.7 (18.0)  GA: 22.2 (16.9)  No difference between PNB and GA group (p=0.821)  Arm & hand:  PNB: 29.7 (32.0)  GA: 29.1 (21.9)  No difference between PNB and GA group (p=0.793)  Mobility  PNB: 6.2 (20.7)  GA: 4.7 (8.8)  No difference between PNB and GA group (p=0.377)  12 weeks: SMFA score (SD), mean  Function:  PNB: 14.1 (12.8)  GA: 18.8 (16.1)  No difference between PNB and GA group (p=0.455)  Bothersome:  PNB: 7.0 (7.9)  GA: 13.6 (23.6)  No difference between PNB and GA group (p=0.388)  Activity:  PNB: 7.7 (10.3)  GA: 13.0 (16.1)  No difference between PNB and GA group (p=0.372)  Emotion:  PNB: 10.4 (11.7)  GA: 14.6 (17.5)  No difference between PNB and GA group (p=0.503)  Arm & hand:  PNB: 10.8 (12.4)  GA: 16.7 (17.5)  No difference between PNB and GA group (p=0.371)  Mobility:  PNB: 2 (5.7)  GA: 3.1 (7.5)  No difference between PNB and GA group (p=0.706) |
| Rundgren (2),  2019 | 6 months: PRWE score (SD), mean  PNB: 17 (19)  GA: 14 (12)  No difference between PNB and GA group (p=0.7) | 6 months: EQ-ED-3L score (0-1*) (SD), mean  PNB: 0.9 (0.1)  GA: 0.9 (0.2)  No difference between PNB and GA group (p=0.7)  **EQ-5D-3L was transformed into an index number with a range of 0 to 1, where 0 is the worst possible and 1 is the best possible* |
| Wong (3), 2020 | 3 months: QuickDASH score (IQR), median  PNB: 34.1 (10.8-50)  GA:21.6 (11.4-47.7)  No difference between PNB and GA group (p=0.839)  6 months: QuickDASH score (IQR), median  PNB: 13.6 (9.1-24.4)  GA: 22.5 (11.4-32.3)  No difference between PNB and GA group (p=0.276) | 3 months: PRWE score (IQR), median  PNB: 35 (11.4-46.6)  GA: 23 (11.9-40.6)  No difference between PNB and GA group (p=0.618)  6 months: PRWE score (IQR), median  PNB: 18.8 (4.1-34.4)  GA: 12 (7-34.8)  No difference between PNB and GA group (p=0.959) |
| **Observational studies** | | |
| Egol (4), 2012 | 3 months: DASH score (SD), mean  PNB: 18.4 (19.6)  GA: 26.3 (27.6)  PNB is superior to GA group (p=0.04)  6 months: DASH score (SD), mean  PNB: 10.2 (18.2)  GA: 17.8 (20.7)  PNB is superior to GA group (p=0.02)  12 months: DASH score (SD), mean  PNB: 11.0 (20.5)  GA: 12.0 (18.0)  No difference between PNB and GA group (p=0.72) | |
| Egol (5), 2014 | 12 months: DASH score (SD), mean  PNB: 38.6  GA: 53.1  PNB is superior to GA group (p=0.003) | |
| Doo (6), 2020 | Pre-operatively: QoR-40K (IQR), median  PNB: 182.5 (172.8-193.5)  GA: 177.0 (166.0-191.0)  No difference between PNB and GA group (p=0.201)  PO day 1: QoR-40K (IQR), median  No difference between PNB and GA group (p=0.158)  PNB: 175.0 (158.0-186.5)  GA: 169 (143.0-185.0)  PO day 7: QoR-40K (IQR), median  PNB: 189.0 (182.8-196.4)  GA: 181.5 (171.3- 196.0)  No difference between PNB and GA group (p=0.207) | |

Legend:

DASH: Disabilities of the Arm, Shoulder and Hand

EQ-ED-3L: EuroQol-5 Dimensions-3

GA: General Anesthesia

IQR: Interquartile Range

N/A: Not Available

PNB: Peripheral Nerve Block

PO: Postoperative

PWRE: Patient Rated Wrist Evaluation

QoR-40K: Quality of Recovery – 40 Korean

QuickDASH: Quick Disabilities of the Arm, Shoulder and Hand

RCT: Randomized Control Trial

SD: Standard Difference

SMFA: Short Musculoskeletal Function Assessment

**Table S6: Psychometric questionnaires**

| **Name** | **Assessment** | **Number of questions** | **Scoring** | **Psychometrics propreties** | **Studies using psychometric questionnaires** |
| --- | --- | --- | --- | --- | --- |
| DASH (7–10) | Disabilities of:  -Shoulder  -Arm  -Hand | 30 | Each question: score between 1-5  1: no difficulty  5: unable  Scoring:  $\frac{\left[ \left( sum of n reponses \right)-1 \right]}{n} \times25$  Final score: 0-100  0: No disabilities  100: maximum disabilities | Validated: yes  Self-reported: yes  Standardized: no | Egol.(4), 2012  Egol (5), 2014  Galos (1),2016 |
| EQ-ED-3L (11) | Mobility  Self-care  Usual activities  Pain/discomfort  Anxiety/depression | 6 | Each question score: 1-3  1: no problem  2: some problem  3: extreme problem  VAS: 0-100  0: the worst  100: the best  Scoring: 5-digit score  Final score  11111: good function  33333: worst function | Validated: yes  Self-reported: yes  Standardized: no | Rundgren (2), 2019 |
| Global QoR-40K (12) | Physical comfort  Emotional state  Psychological support  Physical independence  Pain | 40 | Each question: 1-5  1: none of the time (very poor)  2: all the time (excellent)  Scoring :  Sum of all the answer  Final score : 40-200  40 : extremely poor quality of recovery  200 : excellent quality of recovery | Validated: yes  Self-reported: yes  Standardized: no | Doo (6), 2020 |
| PRWE (13–16) | Pain  Functional recovery:  -Usual activities  -Specific activities | 15 | Each question score: 0-10  0: no pain/ no difficulty  10: worst pain/unable to do task  Pain questions:5  Functional question: 10  Scoring:  Pain subscore:0-50  Functional subscore (total/ by 2): 0-50  Final score: 0-100  0: No disabilities  100: maximum disabilities | Validated: yes  Self-reported: yes  Standardized: no | Rundgren. (2), 2019  Wong (3), 2020 |
| QuickDASH (17–19) | Disabilities of:  -Shoulder  -Arm  -Hand | 11 | Each question: score between 1-5  1: no difficulty  5: unable  Scoring:  $\frac{\left[ \left( sum of n reponses \right)-1 \right]}{n} \times25$  Final score: 0-100  0: No disabilities  100: maximum disabilities | Validated: yes  Self-reported: yes  Standardized: no | Wong (3), 2020 |
| SMFA (20) | Function  Bothersome  Daily activities  Emotion  Arm & hand function  Mobility | 46 | Each question: score between 1-5  1: not at all difficult/none of the time/not bothered  5: unable to do/all of the time/extremely bothered  Scoring:  Daily activities  $\frac{\left[ \left( raw summed score daily activities \right)-10 \right]}{40} \times100$  Emotional status  $\frac{\left[ \left( raw summed score emotional status \right)-7 \right]}{28} \times100$  Arm and Hand Function  $\frac{\left[ \left( raw summed score arm \& hand \right)-8 \right]}{32} \times100$  Mobility  $\frac{\left[ \left( raw summed score mobilty items \right)-9 \right]}{36} \times100$  Function index  $\frac{\left[ \left( raw summed score items 1 to 34 \right)-34 \right]}{136} \times100$  Bothersome index  $\frac{\left[ \left( raw summed score items 35 to 46 \right)-12 \right]}{48} \times100$  Final score: 0-100  0: good function  100: poor function | Validated: yes  Self-reported: yes  Standardized: no | Galos (1), 2016 |

Legend:

DASH: Disabilities of the Arm, Shoulder and Hand

EQ-ED-3L : EuroQol-5 Dimensions-3

PWRE: Patient Rated Wrist Evaluation

QoR-40K: Quality of Recovery – 40 Korean

QuickDASH: Quick Disabilities of the Arm, Shoulder and Hand

SMFA: Short Musculoskeletal Function Assessment

VAS: Visual Analog Scale

**Table S7:ROM at individual timeframe**

| **1^st^ Author, Year** | **ROM** | | | |
| --- | --- | --- | --- | --- |
| **RCT** | | | | |
| Rundgren (2) | 6 months: ROM* (SD), mean  PNB: 0.9 (0.1)  GA: 0.9 (0.1)  No difference between PNB and GA group (p=0.7)  **The ROM was a sum of wrist flexion, extension, ulnar deviation radial deviation, supination and pronation on the injured and non-uninjured side.* | | | |
| **Observational studies** | | | | |
| Egol (4),2012 | *3 MONTHS:* | | | |
|  | ROM (SD), degrees | PNB | GA | Conclusion |
|  | Extension | 50.5 (19.6) | 45.6 (16.8) | PNB is superior to GA group (p=0.09) |
|  | Flexion | 48.5 (16.8) | 40.0 (14.8) | PNB is superior to GA group (p=0.0006) |
|  | Supination | 75.5 (15.0) | 64.4 (24.6) | PNB is superior to GA group (p=0.002) |
|  | Pronation | 80.5 (14.2) | 76.7 (12.9) | **No** difference between PNB and GA group (p=0.08) |
|  | Ulnar deviation | 26.9 (10.2) | 23.0 (11.2) | PNB is superior to GA group (p=0.04) |
|  | Radial deviation | 17.1 (9.4) | 15.8 (14.4) | **No** difference between PNB and GA group (p=0.55) |
|  | Index finger TAM | 251.4 (28.5) | 232.3 (47.9) | PNB is superior to GA group (p=0.005) |
|  | Middle finger TAM | 252.0 (28.2) | 236.1 (44.9) | PNB is superior to GA group (p=0.01) |
|  | Ring finger TAM | 252.9 (28.0) | 235.4 (47.7) | PNB is superior to GA group (p=0.01) |
|  | Little finger TAM | 251.4 (29.7) | 235.0 (43.4) | PNB is superior to GA group (p=0.01) |
|  | Thumb TAM | 151.8 (34.0) | 146.7 (41.6) | **No** difference between PNB and GA group (p=0.43) |
|  | Index finger DPC | 0.16 (0.58) | 0.94 (1.8) | PNB is superior to GA group (p=0.002) |
|  | Middle finger DPC | 0.16 (0.58) | 0.87 (1.9) | PNB is superior to GA group (p=0.005) |
|  | Ring finger DPC | 0.13 (0.49) | 0.90 (1.9) | PNB is superior to GA group (p=0.003) |
|  | Little finger DPC | 0.15 (0.51) | 0.79 (1.6) | PNB is superior to GA group (p=0.004) |
|  | *6 MONTHS:* | | | |
|  | ROM (SD), degrees | PNB | GA | Conclusion |
|  | Extension | 56.9 (19.3) | 50.4 (14.8) | PNB is superior to GA group (p=0.02) |
|  | Flexion | 52.4 (17.7) | 46.8 (14.3) | PNB is superior to GA group (p=0.04) |
|  | Supination | 78.5 (11.7) | 73.9 (18.0) | **No** difference between PNB and GA group (p=0.1) |
|  | Pronation | 82.7 (7.2) | 78.8 (13.0) | PNB is superior to GA group (p=0.04) |
|  | Ulnar deviation | 28.5 (8.5) | 25.7 (10.3) | **No** difference between PNB and GA group (p=0.11) |
|  | Radial deviation | 19.6 (9.3) | 15.5 (6.7) | PNB is superior to GA group (p=0.004) |
|  | Index finger TAM | 258.5 (7.9) | 247.9 (26.6) | PNB is superior to GA group (p=0.005) |
|  | Middle finger TAM | 258.5 (7.9) | 249.9 (22.6) | PNB is superior to GA group (p=0.008) |
|  | Ring finger TAM | 258.5 (7.9) | 248.3 (30.8) | PNB is superior to GA group (p=0.02) |
|  | Little finger TAM | 258.5 (7.9) | 247.3 (33.4) | PNB is superior to GA group (p=0.02) |
|  | Thumb TAM | 163.1 (27.2) | 146.2 (39.0) | **No** difference between PNB and GA group (p=0.26) |
|  | Index finger DPC | 3.1 (2.0) | 2.3 (1.8) | **No** difference between PNB and GA group (p=0.83) |
|  | Middle finger DPC | 0.05 (0.2) | 0.35 (0.9) | PNB is superior to GA group (p=0.02) |
|  | Ring finger DPC | 0.05 (0.2) | 0.37 (1.0) | PNB is superior to GA group (p=0.02) |
|  | Little finger DPC | 0.05 (0.2) | 0.38 (1.1) | PNB is superior to GA group (p=0.02) |
|  | *12 MONTHS:* | | | |
|  | ROM (SD), degrees | PNB | GA | Conclusion |
|  | Extension | 58.6 (22.1) | 52.8 (13.6) | PNB is superior to GA group (p=0.03) |
|  | Flexion | 56.0 (19.1) | 49.8 (12.9) | PNB is superior to GA group (p=0.01) |
|  | Supination | 79.3 (10.9) | 80.2 (12.4) | **No** difference between PNB and GA group (p=0.64) |
|  | Pronation | 83.7 (5.2) | 83.2 (5.9) | **No** difference between PNB and GA group (p=0.6) |
|  | Ulnar deviation | 29.6 (9.2) | 29.1 (9.0) | **No** difference between PNB and GA group (p=0.79) |
|  | Radial deviation | 22.5 (9.6) | 20.6 (12.5) | **No** difference between PNB and GA group (p=0.33) |
|  | Index finger TAM | 260.0 (<0.001) | 255.4 (17.2) | PNB is superior to GA group (p=0.04) |
|  | Middle finger TAM | 259.6 (2.6) | 257.1 (12.9) | **No** difference between PNB and GA group (p=0.15) |
|  | Ring finger TAM | 259.6 (2.6) | 270.0 (12.4) | **No** difference between PNB and GA group (p=0.12) |
|  | Little finger TAM | 260.0 (<0.001) | 255.0 (19.5) | **No** difference between PNB and GA group (p=0.06) |
|  | Thumb TAM | 164.7 (19.5) | 157.6 (30.3) | **No** difference between PNB and GA group (p=0.11) |
|  | Index finger DPC | 0.46 (0.4) | 0.19 (0.6) | PNB is superior to GA group (p=0.08) |
|  | Middle finger DPC | 0.01 (0.04) | 0.16 (0.7) | PNB is superior to GA group (p=0.05) |
|  | Ring finger DPC | 0.01 (0.04) | 0.18 (0.7) | **No** difference between PNB and GA group (p=0.16) |
|  | Little finger DPC | 0 (<0.001) | 0.12 (0.5) | **No** difference between PNB and GA group (p=0.06) |
| Egol (5), 2014 | 12 months: ROM degrees  Forward elevation:  PNB: 139  GA: 119  PNB is superior to GA group (p=0.002)  Passive forward elevation:  PNB: 147  GA: 131  PNB is superior to GA group (p=0.005)  External rotation:  PNB: 50  GA: 40  PNB is superior to GA group (p=0.002)  Internal rotation:  No difference between (p=0.51) | | | |

Legend:

DPC: Distal Palmar Cease

GA: General Anesthesia

PNB: Peripheral Nerve Block

RCT: Randomized Control Trial

ROM: Range of Motion

SD: Standard Deviation

TAM: Total Active Mouvement

**REFERENCES**

1. Galos DK, Taormina DP, Crespo A, Ding DY, Sapienza A, Jain S, et al. Does Brachial Plexus Blockade Result in Improved Pain Scores After Distal Radius Fracture Fixation? A Randomized Trial. Clin Orthop. mai 2016;474(5):1247‑54.

2. Rundgren J, Mellstrand Navarro C, Ponzer S, Regberg A, Serenius S, Enocson A. Regional or General Anesthesia in the Surgical Treatment of Distal Radial Fractures: A Randomized Clinical Trial. J Bone Joint Surg Am. 2019;101(13):1168‑76.

3. Wong J, Chung F. Development of the Functional Recovery Index for Ambulatory Surgery and Anesthesia. 2009;110(3):7.

4. Egol KA, Soojian MG, Walsh M, Katz J, Rosenberg AD, Paksima N. Regional anesthesia improves outcome after distal radius fracture fixation over general anesthesia. J Orthop Trauma. sept 2012;26(9):545‑9.

5. Egol KA, Forman J, Ong C, Rosenberg A, Karia R, Zuckerman JD. Regional Anesthesia Improves Outcome in Patients Undergoing Proximal Humerus Fracture Repair. Bull NYU Hosp Jt Dis N Y. 2014;72(3):231‑6.

6. Doo AR, Kang S, Kim YS, Lee TW, Lee JR, Kim DC. The effect of the type of anesthesia on the quality of postoperative recovery after orthopedic forearm surgery. Korean J Anesthesiol. 2020;73(1):58‑66.

7. dash_scoring_2010.pdf [Internet]. [cité 20 nov 2018]. Disponible sur: http://www.dash.iwh.on.ca/sites/dash/files/downloads/dash_scoring_2010.pdf

8. Beaton DE, Katz JN, Fossel AH, Wright JG, Tarasuk V, Bombardier C. Measuring the whole or the parts? J Hand Ther. avr 2001;14(2):128‑42.

9. Gummesson C, Atroshi I, Ekdahl C. The disabilities of the arm, shoulder and hand (DASH) outcome questionnaire: longitudinal construct validity and measuring self-rated health change after surgery. BMC Musculoskelet Disord. 16 juin 2003;4(1):11.

10. SMET LD. The DASH questionnaire and score in the evaluation of hand and wrist disorders. 2008;74:7.

11. EQ-5D-3L – EQ-5D [Internet]. [cité 14 oct 2021]. Disponible sur: https://euroqol.org/eq-5d-instruments/eq-5d-3l-about/

12. Lee JH, Kim D, Seo D, Son J seon, Kim DC. Validity and reliability of the Korean version of the Quality of Recovery-40 questionnaire. Korean J Anesthesiol. déc 2018;71(6):467‑75.

13. Smith MV, Calfee RP, Baumgarten KM, Brophy RH, Wright RW. Upper Extremity-Specific Measures of Disability and Outcomes in Orthopaedic Surgery: J Bone Jt Surg-Am Vol. févr 2012;94(3):277‑85.

14. Kleinlugtenbelt YV, Krol RG, Bhandari M, Goslings JC, Poolman RW, Scholtes VAB. Are the patient-rated wrist evaluation (PRWE) and the disabilities of the arm, shoulder and hand (DASH) questionnaire used in distal radial fractures truly valid and reliable? Bone Jt Res. 8 févr 2018;7(1):36‑45.

15. MacDermid JC, Turgeon T, Richards RS, Beadle M, Roth JH. Patient rating of wrist pain and disability: a reliable and valid measurement tool. J Orthop Trauma. déc 1998;12(8):577‑86.

16. De Smet LL. The DASH questionnaire and score in the evaluation of hand and wrist disorders. Acta orthopaedica Belgica. 74(5). :575.

17. quickdash_info_2010.pdf [Internet]. [cité 20 nov 2018]. Disponible sur: http://www.dash.iwh.on.ca/sites/dash/files/downloads/quickdash_info_2010.pdf

18. Mintken PE, Glynn P, Cleland JA. Psychometric properties of the shortened disabilities of the Arm, Shoulder, and Hand Questionnaire (QuickDASH) and Numeric Pain Rating Scale in patients with shoulder pain. J Shoulder Elbow Surg. 1 nov 2009;18(6):920‑6.

19. Beaton DE, Wright JG, Katz JN. Development of the QuickDASH: Comparison of Three Item-Reduction Approaches. J Bone Jt Surg. mai 2005;87(5):1038‑46.

20. Swiontkowski MF, Engelberg R, Martin DP, Agel J. Short musculoskeletal function assessment questionnaire: validity, reliability, and responsiveness. J Bone Joint Surg Am. sept 1999;81(9):1245‑60.
